# Supplementary material for: Food handlers’ knowledge, attitudes and self-reported practices regarding safe food handling in charitable food assistance programmes in the eThekwini District, South Africa: cross-sectional study
Source: BMJ Open. 2023 Apr 25;13(4):e065357. doi: 10.1136/bmjopen-2022-065357 (PMC10151870; doi:10.1136/bmjopen-2022-065357)
Supplement: Author's manuscript [file bmjopen-2022-065357.draft_revisions.pdf]

BMJ Open is committed to open peer review. As part of this commitment we make the peer review history of every article we publish publicly available.

When an article is published we post the peer reviewers' comments and the authors' responses online. We also post the versions of the paper that were used during peer review. These are the versions that the peer review comments apply to.

The versions of the paper that follow are the versions that were submitted during the peer review process. They are not the versions of record or the final published versions. They should not be cited or distributed as the published version of this manuscript.

BMJ Open is an open access journal and the full, final, typeset and author-corrected version of record of the manuscript is available on our site with no access controls, subscription charges or pay-per-view fees (http://bmjopen.bmj.com).

If you have any questions on BMJ Open's open peer review process please email info.bmjopen@bmj.com

BMJ Open

Food handlers' knowledge, attitudes, and self-reported practices of safe food handling in charitable food assistance programs in the eThekwini District, South Africa

Journal:	*BMJ Open*
Manuscript ID	bmjopen-2022-065357
Article Type:	Original research
Date Submitted by the Author:	02-Jun-2022
Complete List of Authors:	Makhunga, Sizwe; University of KwaZulu-Natal College of Health Sciences, Public Health<br>Macherera, Margaret; Lupane State University, Department of Crop and Soil Sciences<br>Hlongwana, Khumbulani; University of KwaZulu-Natal, Department of Public Health Medicine; University of KwaZulu-Natal
Keywords:	Nutritional support < GASTROENTEROLOGY, Health & safety < HEALTH SERVICES ADMINISTRATION & MANAGEMENT, Health policy < HEALTH SERVICES ADMINISTRATION & MANAGEMENT, Public health < INFECTIOUS DISEASES

SCHOLARONE™
Manuscripts

I, the Submitting Author has the right to grant and does grant on behalf of all authors of the Work (as defined in the below author licence), an exclusive licence and/or a non-exclusive licence for contributions from authors who are: i) UK Crown employees; ii) where BMJ has agreed a CC-BY licence shall apply, and/or iii) in accordance with the terms applicable for US Federal Government officers or employees acting as part of their official duties; on a worldwide, perpetual, irrevocable, royalty-free basis to BMJ Publishing Group Ltd ("BMJ") its licensees and where the relevant Journal is co-owned by BMJ to the co-owners of the Journal, to publish the Work in this journal and any other BMJ products and to exploit all rights, as set out in our licence.

The Submitting Author accepts and understands that any supply made under these terms is made by BMJ to the Submitting Author unless you are acting as an employee on behalf of your employer or a postgraduate student of an affiliated institution which is paying any applicable article publishing charge ("APC") for Open Access articles. Where the Submitting Author wishes to make the Work available on an Open Access basis (and intends to pay the relevant APC), the terms of reuse of such Open Access shall be governed by a Creative Commons licence – details of these licences and which Creative Commons licence will apply to this Work are set out in our licence referred to above.

Other than as permitted in any relevant BMJ Author's Self Archiving Policies, I confirm this Work has not been accepted for publication elsewhere, is not being considered for publication elsewhere and does not duplicate material already published. I confirm all authors consent to publication of this Work and authorise the granting of this licence.

For peer review only

Food handlers' knowledge, attitudes, and self-reported practices of safe food handling in charitable food assistance programs in the eThekwini District, South Africa

Sizwe Makhunga[1], Margaret Macherera[2], and Khumbulani Hlongwana[1,3]

[1]Department of Public Health Medicine, School of Nursing and Public Health, University of KwaZulu-Natal, Durban, South Africa

[2]Department of Crop and Soil Sciences, Faculty of Agricultural Sciences, Lupane State University, Lupane, Zimbabwe

[3]Cancer and Infectious Diseases Epidemiology Research Unit (CIDERU), School of Nursing and Public Health, University of KwaZulu-Natal, Durban, South Africa

Corresponding Author: Sizwe Makhunga (Ph.D. Candidate)

Address: 2nd Floor George Campbell Building, Department of Public Health Medicine, School of Nursing and Public Health, University of KwaZulu-Natal, Durban, 4001, South Africa

Email address: makhungas@gmail.com/ 951030918@stu.ukzn.ac.za

Tel: +27730623901/ +27313229852

Email addresses of authors

Khumbulani Hlongwana (Hlongwanak@ukzn.ac.za)

Margaret Macherera (mmacherera@gmail.com)

For peer review only

Abstract

Objective: This study aimed to assess food handlers' knowledge, attitudes, and self-reported practices towards the safer donated foods.

Design This cross-sectional study was conducted from January to May 2021.

Setting: This study was conducted in the eThekwini District, South Africa.

Participants: A criterion-based sampling technique and the exponential non-discriminative snowball sampling methods were used to identify the CFAPs. The principal investigator administered the validated English or isiZulu structured Questionnaire adapted from the World Health Organization's (WHO) five keys to a safer food manual.

Primary and secondary outcome measures: The primary outcome was the knowledge, attitudes, and self-reported practices towards the safer donated foods. The secondary outcomes were walkthrough surveys of infrastructure and observations of practices.

Results: A total of 252 food handlers from sixty-three CFAPs participated. A minimum of one food handler and a maximum of six food handlers were recruited in each of the 63 participating CFAPs. While most of the food handlers (87,7%) had no formal prior safe food handling training, 72,2% had overall work experience of more than ten years, and 60,7% of food handlers worked in the food service industry all their lives. About 85% of respondents knew the risk of food cross-contamination.

Conclusions: Despite food handlers' extensive working experience in charitable food assistance practice, several gaps were identified, implying that experience alone is insufficient for correct practices.

Keywords: Knowledge, Attitudes, Self-reported practices, Food handlers, Charitable food assistance programs

Strengths and Limitations of this Study

- The study's key strength is the lack of similar studies published in a South African setting.
- The sample size of the study was not too large, however, the diversity of CFAPs from which the sample was drawn offers unique strength to the study.
- The study was prone to social desirability bias, given that most of the data emanated from self-reporting.

For peer review only - http://bmjopen.bmj.com/site/about/guidelines.xhtml

- The current findings can be used to support the promotion of food handler training, particularly in CFAPs.

Introduction

Foodborne diseases (FBDs) resulting from contaminated food continue to pose significant public health challenges worldwide, particularly in low and middle-income countries (LMICs), where socio-economic inequities are pervasive (1). FBDs are a diverse group of illnesses caused by eating foods contaminated with infectious or poisonous microbes and/or chemicals (2). Pathogenic microorganisms, such as bacteria, viruses, parasites, fungus, and chemicals, are the primary causes of FBDs (3). In the clinical environment, FBDs are most exhibited as gastrointestinal symptoms. Other symptoms, such as neurological, immunological, and gynaecological issues, may also occur (2).

While unsafe food is a global health hazard, infants, small children, the elderly, pregnant women, and persons with underlying illnesses are most susceptible, putting them at greater risk for severe FBD (4-6). FBDs are disproportionately distributed globally, with Africa having the highest rate of FBD per capita globally. Infections associated with diarrhoeal diseases account for a significant proportion of the cases (4). The high incidence of FBD is due to the usage of unfit water for cleaning and processing food, inefficient food production, poor food handling, insufficient food-storage infrastructure, and regulatory policies that are either ineffective or ineffectively enforced (7-9). The listeriosis outbreak of 2017 to 2018 in South Africa was a reminder of the potentially catastrophic consequences of the FBD outbreak, underscoring the need to improve food safety controls and interventions (1) and (3). Nine hundred and thirty-seven cases were found, 465 (50%) of which were linked to pregnancy, and 406 (87%) of the pregnancy-related cases occurred in neonates. Two hundred and twenty-nine (24%) of the 937 cases involved individuals aged 15 to 49, excluding those who were pregnant. Among the patients whose HIV status was known, 38% of those with pregnancy-related cases (77 of 204) and 46% of the remaining patients (97 of 211) were infected with the listeria virus. One hundred and ninety-three (27%) of the 728 patients with a known outcome died (10-12).

In KwaZulu-Natal (KZN), the health risks associated with unsafe food handling are prevalent, owing to the high incidence of TB, HIV, and AIDS in the province (13, 14). Research has

For peer review only - http://bmjopen.bmj.com/site/about/guidelines.xhtml

shown that a seemingly minor foodborne infection can be lethal in immunocompromised people (15). According to the National Institute for Communicable Diseases (NICD) Review Report on FBD outbreaks, the KwaZulu-Natal province had the most (5 cases per 100 000 population) FBD outbreaks between March 2018 and March 2020, outnumbering all nine provinces in South Africa. (7). Research has shown that malnutrition and diarrhoea caused by consuming contaminated food are the leading causes of infant mortality in hygiene-challenged settings like the eThekwini District (16).

Fifty-four percent (54%) of the unemployed in the KZN province live in the eThekwini district, thereby making the district vulnerable to health conditions exacerbated by poverty. For example, 31% of the population in the eThekwini district is poor, putting the district at increased risk of food insecurity (14). As a result, there is a strong reliance on government services and charitable food assistance programs (CFAPs). Food outlets that collect and distribute surplus food to food-insecure persons are (CFAPs) (10, 11). Food banks, food rescue organizations, pantries, community soup kitchens, child and youth care centers, and emergency shelters are among these organizations (10, 12). Despite several CFAPs operating in the eThekwini district for decades, there is a lack of data about the status of their safety and hygiene practices. Studies, however, have shown that the majority of CFAP food handlers are unskilled and operate on a volunteer basis (17).

Food handlers' knowledge, attitudes, and practices toward safe food handling are essential in improving food safety and reducing inadvertent foodborne illnesses (5, 18). Food contamination caused by poor personal hygiene, particularly inefficient hand washing, has been identified as an essential risk factor for food poisoning (19). Unlike in the conventional food supply chain, there is a lack of data on hygiene practices regarding food handling in CFAPs (17, 20). Thus, this study investigated food handlers' knowledge, attitudes, and self-reported practices of food handling on five keys to safer food, based on the World Health Organization's (WHO) five keys to a safer food manual (21, 22) in the eThekwini District's CFAPs in South Africa. The results of this study can be used to inform the strategies for improving food safety in CFAPs and ultimately protect vulnerable populations from foodborne diseases.

For peer review only - http://bmjopen.bmj.com/site/about/guidelines.xhtml

Materials and methods

The study area

This study was conducted in eThekwini District, South Africa, between January and May 2021. EThekwini District is one of the eleven Districts in KwaZulu-Natal (KZN), South Africa. It comprises 103 wards that are urban, rural, and peri-rural in nature, which spread across eight sub-districts: South Central, South-West, Umlazi and Engonyameni, Lower South, North Central, Greater Inanda /Tongaat Sub-District, Inner-West, and Outer West. It covers approximately 2,556 km^2 area stretching from the Umkomaas in the south, Tongaat in the north, and Cato Ridge in the west, including a few tribal areas in Umbumbulu, and Ndwedwe. It has an estimated population of 3,702,231 (2016 estimate) and a population density of 1,448.5 people per km^2, with a sizeable population settling in the South Region (41%), followed by the North Region (32%) and the West Region (27%) (14).

Despite being highly urbanized and densely populated, pockets of rural communities exist on the west, south, and north outskirts, impacting equitable access to essential services (14).

Research design and sampling

A criterion-based sampling technique was used to identify CFAPs. This was predominantly achieved through word-of-mouth using the few local CFAPs known to the researcher and the cohort identified from the National Department of Social Department (NDSD) website. Those initial CFAPs referred the researcher to other CFAP networks as part of the exponential non-discriminative snowball sampling methods (23, 24). The snowballing continued until enough CFAPs (n=252) were reached, guided by the statistical conclusions, suggesting a minimum of 192 CFAPs. All participants recruited into this study were strictly staff members who had direct contact with food during receiving, checking and storage, preparation, processing/cooking, serving, packaging, and distribution, or washing and cleaning the food facility.

Data collection

A researcher administered the structured questionnaire face-to-face to all sampled and consenting food handlers (n=252) in the selected CFAPs (n=63). A minimum of one food handler and a maximum of six food handlers were recruited in each of the 63 participating CFAPs. The selected CFAPs were visited during their regular operating hours. Each participant signed an informed consent form before participating in the study. The inclusion criteria were all persons involved in food handling. The questionnaire was pilot tested in ten CFAPs at the

For peer review only - http://bmjopen.bmj.com/site/about/guidelines.xhtml

Umsunduzi Municipality in Pietermaritzburg, South Africa, and the information obtained was not included in the final analysis.

Research instrument

The questionnaire was adapted from the World Health Organization's 'Five keys to safer food' guideline for food handlers (22). We used the manual of the five keys available on the WHO site, first released in a poster in 2001. This handbook includes two sections: section one provides background information for the manual, and section two stipulates five keys to safer food. Three forms at the end of section two of the handbook were used to assess the participants' food safety knowledge, attitude, and self-reported behavior. The questionnaire was structured into six distinctive parts to collect information (i) demographic characteristics, (ii) background characteristics of the CFAPs, (iii) food hygiene & safety practices and general requirements, (iv) knowledge questions, (v) attitude questions, and (vi) self-reported practices. However, this paper focuses on four parts, thereby excluding parts two and three. We also used a checklist to gather data during walkthrough surveys and observations of practices.

Statistical analysis

All statistical analyses were performed using StataCorp. 2021. *Stata Statistical Software: Release 17*. College Station, TX: StataCorp LLC. We used descriptive statistics to summarize the CFAPs and food-services staff's general characteristics and describe their food hygiene and safety knowledge, attitudes, and self-reported practices. By categorizing answers for each section as dichotomous variables, statistical analysis of the relationship between questionnaire answers and demographic characteristics of respondents was performed: knowledge was classified and recorded as correct vs. incorrect, attitudes as agreement vs. disagreement/uncertain, and practices as safe, when an answer was always, vs. unsafe, when an answer was often or never.

Pearson's chi-square test was used to calculate the frequency analysis results of respondents' food safety knowledge, attitude, and practice, and only the questions with a proportion of accurate answers of 95 percent or less were reported. The level of knowledge of the food handlers was determined through the following criteria, namely: each correct response received one point, while each incorrect answer received zero points. The score allocation for attitude was two points for agreeing, one point for not sure, and a zero for disagree. Based on the response to 11 knowledge questions, the scores could vary from 0 to 11. Three levels were

For peer review only - http://bmjopen.bmj.com/site/about/guidelines.xhtml

considered for this: scores between 0 to 4 were classified as poor, 5 to 7 as moderate, and 8 to 11 as good. Total scores could differ for ten attitude questions, and 0 to 20 classifications were allocated: 0 to 6 being negative, 7 to 13 being neutral, and 14 to 20 being positive attitude levels. For ten practice questions, participants could choose from options 1 to 5, where one referred to never and 5 to always. Scores were allocated as follows: always (4), mostly (3), sometimes (2), rarely (1), and never (0).

Ethical Considerations

The ethical clearance (BREC/00000821/2019) was obtained from the University of KwaZulu-Natal Biomedical Research Ethics Committee (BREC). The eThekwini District approved the study, and all participants consented to participate in the study.

Patient and public involvement

Patients and/or the public were not involved in the design, or conduct, or reporting, or dissemination plans of this research.

Results

Distribution of study participants among sub-districts

A total of 252 food handlers participated, with most coming from the Greater Inanda/Tongaat Sub-District and Lower South (**Table 1**).

Table 1: Distribution of participating CFAPs (n = 63) and study participants (n = 252)

Location	CFAPs (n=63)	Food handlers (n=252)
South Central	6	40
South-West	6	18
Umlazi and Engonyameni	10	20
Lower South	9	50
North Central	9	40
Greater Inanda /Tongaat Sub-District	14	70
Inner West	3	8
Outer West	6	6

For peer review only - http://bmjopen.bmj.com/site/about/guidelines.xhtml

Sociodemographic characteristics of the food handlers

Ninety-four percent (n = 237/252) of the food handlers were females, with 55,2% over 41 years (**Table 2**). Less than half () of food handlers had a Participants with Grade 12 (Matric) certification and post-matric qualification constituted 40,5% and 59,1%, respectively. The majority (72,2%) of participants had more than ten years of service experience, 60,7% of which was related to the foodservice industry. All food handlers (100%) had direct contact with food during receiving, checking, and storage, and as high as 80% of them were involved in food preparation and processing/cooking. The majority (88,1%) of participants reported their primary role as serving, packaging & distribution, with only 14,7% washing and cleaning the food facility. Only 12,3 percent of food handlers had attended at least one official training session on food hygiene elements within five years of the research, while the majority (87,7%) had had no training in food safety (**Table 2**).

Table 2: Demographic characteristics of food handlers (n = 252)

Socio demographic characteristics	Frequency (n)	Percentage (%)
**Gender**		
Male	15	6,0
Female	237	94,0
**Age group (years)**		
20 – 29	14	5,5
30 – 39	36	14,3
40 – 49	78	31,0
50 +	124	49,2
**Race**		
Black	144	57,1
White	19	7,5
Indian	69	27,4
Coloured	20	7,9
**Marital Status**		
Single	106	42,1
Married	107	42,5
Divorced	29	11,5
Widowed	10	4,0
**Number of dependents**		
None	24	9,5
<2	67	26,6
2-5	124	49,2
> 5	37	14,7
**Place of residence**		
Formal settlement	181	71,8
Informal settlement	3	1,2
Shelter	68	27,0

For peer review only

For peer review only - http://bmjopen.bmj.com/site/about/guidelines.xhtml

**Education level**		
Primary	1	0,4
Secondary	102	40,5
Tertiary	149	59,1
**Overall work experience (years)**		
3-5	18	7,1
6-10	52	20,6
> 10	182	72,2
**Experience in foodservice (years)**		
< 1	6	2,4
1-3	23	9,1
4-6	53	21,0
7-10	17	6,7
> 10	153	60,7
**Main Job responsibility**		
Food receiving, checking & storage	252	100,0
Food preparation, process/cooking	202	80,2
Food serving/packaging/distribution	222	88,1
Washing and cleaning	37	14,7
Other	0	0,0
**Attended safe food handling training**		
Yes	31	12,3
No	221	87,7
If yes, date (years)		
≤ 2	17	6,7
3-4	14	5,6
N/A	221	87,7

Knowledge

All food handlers (100%) knew that it is essential to wash their hands before handling food (**Table 3**). About 85% of food handlers were aware of the dangers of food cross-contamination and consequently reported that they had taken steps to avoid it. However, 17% of food handlers were unaware that wiping cloths can spread bacteria, primarily when used filthy. Most food handlers (71%) were enthusiastic about safe food handling. However, about 53% admitted to never properly reheating cooked meals when asked about their food handling methods. Most (92,5%) of the food handlers did not appreciate the importance of thorough cooking and temperature control. Although almost all food handlers (99,6%) reported that they wash fruits and vegetables, the majority (90,7%) thought that one could tell the safety of water by simply looking at it.

Table 3: Food handlers' knowledge of safe food handling in charitable food assistance programs (n = 252)

For peer review only

Statement/Question		True No (%)	False No (%)
**1.**	It is important to wash hands before handling food	252 (100)	0 (0)
**2.**	Wiping cloths can spread microorganisms	208 (82,5)	44 (17,5)
**3.**	The same cutting board can be used for raw and cooked foods provided it looks clean	39 (15,5)	213 (84,5)
**4.**	Raw food needs to be stored separately from cooked food	216 (85,7)	36 (14,3)
**5.**	Cooked foods do not need to be thoroughly reheated	186 (73,8)	66 (26,2)
**6.**	Proper cooking includes meat cooked to 40 °C	233 (92,5)	19 (7,5)
**7.**	Cooked meat can be left at room temperature overnight to cool before refrigerating	62 (24,6)	190 (75,4)
**8.**	Cooked food should be kept very hot before serving	148 (58,7)	104 (41,3)
**9.**	Refrigerating food only slows bacterial growth	154 (61,1)	98 (38,9)
**10.**	Safe water can be identified by the way it looks	229 (90,9)	23 (9,1)
**11.**	Wash fruit and vegetables	251 (99,6)	1 (0,4)

Attitude

All the food handlers (100%) agreed that it is crucial to observe general hygiene, and 89,3% believed that using different knives and cutting boards for raw and cooked foods is worth the extra effort. However, 5,2% did not show appreciation of the importance of guarding against cross-contamination, and 5,5% were uncertain. Almost all food handlers (96,8%) reported that they ensured thorough cooking and observed temperature controls. They were also aware of the consequences of improperly thawing food. Furthermore, 75% of food handlers believe that leaving prepared food out at room temperature for more than two hours without refrigeration is dangerous (**Table 4**). The participants, overall, had a positive attitude.

Table 4: Attitudes of food handlers towards safe food handling in charitable food assistance programs (n = 252)

Statement/Question		Agree No (%)	Unsure No (%)	Disagree No (%)
**1.**	Frequent handwashing during food preparation is worth the extra time	252 (100)	0 (0)	0 (0)
**2.**	Keeping kitchen surfaces clean reduces the risk of illness	252 (100)	0 (0)	0 (0)
**3.**	Keeping raw and cooked food separate helps to prevent illness	225 (89,3)	27 (10,7)	0 (0)
**4.**	Using different knives and cutting boards for raw and cooked foods is worth the extra effort	225 (89,3)	14 (5,5)	13 (5,2)
**5.**	Meat thermometers help ensure food is cooked thoroughly	196 (77,8)	29 (11,5)	27 (10,7)
**6.**	Soups and stews should always be boiled to ensure the safety	244 (96,8)	0 (0)	8 (3,2)

For peer review only

**7.** Thawing food in a cool place is safer	244 (96,8)	8 (3,2)	0 (0)
**8.** I think it is unsafe to leave cooked food out of the refrigerator for more than two hours	189 (75,0)	28 (11,1)	35 (13,9)
**9.** Inspecting food for freshness and wholesomeness is valuable	252 (100)	0 (0)	0 (0)
**10.** I think it is important to throw away foods that have reached their expiry date	252 (100)	0 (0)	0 (0)

Self-reported practices

All food handlers (100%) stated that they always maintain a high level of general hygiene (**Table 5**). Nearly two-thirds (65,9%) of the participants used separate surfaces for cooked and ready-to-eat food. However, 84,9% of the participants reported that they segregated raw and cooked food during storage. Food handlers reported observed temperature control in 68,3 percent and 55,6 percent of cases, respectively. Less than half (43,7%) of the participants said it was necessary to reheat cooked food until it was boiling all over. All food handlers (100%) said they inspect and discard food that has passed its expiration date. They also asserted that they wash fruits and vegetables before consuming them with clean water.

Table 5: Self-reported practices of food handlers in charitable food assistance programs (n = 252)

**Statement/Question**	**Always No (%)**	**Mostly No (%)**	**Sometimes No (%)**	**Rarely No (%)**	**Never No (%)**
**1.** I wash my hands before and during food preparation	252 (100)	0 (0)	0 (0)	0 (0)	0 (0)
**2.** I clean surfaces and equipment used for food preparation before re-using on other food	252 (100)	0 (0)	0 (0)	0 (0)	0 (0)
**3.** I use separate utensils and cutting boards when preparing raw and cooked food	166 (65,9)	59 (23,1)	0 (0)	0 (0)	27 (11)
**4.** I separate raw and cooked food during storage	214 (84,9)	9 (3,6)	0 (0)	0 (0)	29 (12)
**5.** I check that meats are cooked thoroughly by ensuring that the juices are clear or by using a thermometer	217 (86,0)	0 (0)	0 (0)	0 (0)	35 (14)
**6.** I reheat cooked food until it is piping hot throughout	110 (43,7)	6 (2,4)	0 (0)	0 (0)	134 (53)
**7.** I thaw frozen food in the refrigerator or other cool place	140 (55,6)	7 (28)	23 (9,1)	0 (0)	82 (33)
**8.** After I have cooked a meal, I store any leftovers in a cool place within two hours	172 (68,3)	2 (0,8)	57 (22,6)	0 (0)	21 (8)

For peer review only

For peer review only - http://bmjopen.bmj.com/site/about/guidelines.xhtml

**9.** I check and throw away food beyond its expiry date	252 (100)	0 (0)	0 (0)	0 (0)	0 (0)
**10.** I wash fruit and vegetables with safe water before eating them	252 (100)	0 (0)	0 (0)	0 (0)	0 (0)

1.1 Association among the food safety knowledge, attitudes, and practices of food handlers

Significant correlations were found between knowledge and attitudes (p = 0,001), knowledge and practices (p<0,001), and attitudes and practices (p=0,02). However, the correlations between knowledge vs. attitude and attitude vs. practice were poor (spearman's rho < 0,3), and the association between knowledge vs. practice was moderate (0,3 – 0,7). This means that it is impossible to say with certainty whether respondents' food safety knowledge will influence their attitude, though it may influence their practices around food safety. The findings also show that food handlers' attitudes toward food safety do not accurately reflect their actual food safety practices (**Table 6**).

Table 6: *Association among food safety knowledge, attitudes, and practices*

Level	Spearman's rho	Significance
Knowledge vs. Attitude	0,25	0,001
Knowledge vs. Practice	0,43	<0,001
Attitude vs. Practice	0,15	0,02

Discussion

This study showed that food handlers in CFAPs had relatively good knowledge, attitudes, and practices, even though most food handlers (87,7%) lacked training in food safety, with only 12,3% having attended at least one official training course on food safety and general hygiene within five years of the study. Staff retention appeared to be somewhat high, given that 72,2% of food handlers had more than ten years of service experience, 60,7% of which was in the foodservice industry. All food handler (100%) understood the importance of washing their hands before handling food. Although 85% of food handlers knew the risks of food cross-contamination, 17% were unaware that wiping cloths can spread bacteria, mainly when used filthy. Almost all (92,5%) food handlers participating in this study did not realize the importance of thorough cooking and temperature control, as evidenced by 53% of food handlers who admitted to never properly reheating cooked meals. Although almost all food

For peer review only - http://bmjopen.bmj.com/site/about/guidelines.xhtml

handlers (99,6%) reported that they wash fruits and vegetables, the majority (90,7%) thought that one could tell the safety of water by simply looking at it.

According to this study, food handlers in the CFAPs recounted good knowledge, positive attitudes, and good self-reported practices related to safe food handling. The findings are consistent with other studies conducted in the conventional food supply chain in LMICs settings (25-29). In their recent study, Fariba et al. (22) found that food handlers were good at separating raw and cooked materials and using clean water and cloth. Their findings are consistent with ours, which revealed that most (85%) food handlers in CFAPs knew the risks of food cross-contamination. This is most likely due to extensive food handling experience accrued over a long time, as most food handlers had over ten years of experience, especially in the foodservice industry. When interviewed, some food handlers asserted that they had worked for big corporate restaurants before joining the CFAPs as food service staff. Their knowledge and experience can be enhanced and augmented with current and formal routine food safety training. Local authorities can offer support to CFAPs by rendering services such as regular inspections, food sampling, certification/permitting, seizure and disposal of expired foodstuffs, and health and hygiene education and training, as seen in high income countries (30-32).

The current study's findings support prior research (27-30), which reveal that most food handlers in CFAPs are reckless with donated food. This is because while volunteering, no training in proper food handling is provided to food handlers. However, it was discovered that most food handlers (72,2%) had more than ten years of experience, and that 60,7% of the workers had spent the most of their working lives in the foodservice industry. While this may appear to be a good thing in the sense that workers have gained a lot of experience over time, several studies have warned against complacency as the job becomes dull and workers lose interest in their profession. Most food handlers become casual with safe food handling at this point, putting the food at danger of contamination (33-35).

On the other hand, some CFAPs food handlers claimed a lack of basic infrastructure and resources, such as limited workstations, poor food preparation/processing equipment, and inadequate storage facilities, to name but a few. The walkthrough inspections and observations by the researcher also corroborated the reports by some CFAPs food handlers. These challenges make it difficult for CFAP's food handlers to put their knowledge and competence acquired over the years to optimal use. Nevertheless, CFAP food handlers' understanding of proper

For peer review only - http://bmjopen.bmj.com/site/about/guidelines.xhtml

reheating of cooked food, safe water by appearance, food holding temperature, and the danger zone, remained weak. These findings were consistent with the conclusions made by Sharif et al. (36) asserting that despite good knowledge levels among food handlers, they had insufficient knowledge on thawing, thorough cooking, reheating, and safe temperature. Other studies have shown that this could pose potential risks or cause food poisoning in consumers (37-40).

The study's key strength is the lack of similar studies published in South African setting. The sample size of the study was not too large, however, the diversity of CFAPs from which the sample was drawn offers a unique strength to the study. However, our study was prone to social desirability bias, given that most of the data emanated from self-reporting. However, the infusion of walkthrough as part of the data collection technique to complement the survey questionnaire, alongside the comprehensive nature of consent process, served as an important mitigation. Nonetheless, the current findings can be used to support the promotion of food handler training, particularly in CFAPs. Based on our findings, we suggest that future research investigate the perceived barriers and facilitators to food safety and general hygiene in CFAPs.

Conclusion

The results of this study revealed relatively good knowledge, attitude, and practice regarding food safety in CFAPs' food handlers. However, there is a lack of knowledge and self-reported practices on important factors related to thorough cooking and temperature control. These findings have important implications for developing proper and continuous food safety education and training programs in South Africa's CFAPs and other comparable settings. Food handlers in charitable food assistance programs can never rely solely on experience, hence refresher trainings should be part and parcel of continued improvements in food handling. They require regular health and hygiene education and training to enhance and sustain their knowledge, attitude, and self-reported safe food handling practices.

Ethical approval

The ethical clearance BREC/00000821/2019 was applied for and obtained from the University of KwaZulu-Natal Biomedical Research Ethics Committee (BREC). The eThekwini District approved for the study to be conducted, and all participants consented to participate in the study.

Patient and Public Involvement

For peer review only - http://bmjopen.bmj.com/site/about/guidelines.xhtml

Patients and/or the public were not involved in the design, or conduct, or reporting, or dissemination plans of this research.

Statements

Acknowledgments

IREX funding through the UASP alumni small grants program is acknowledged for providing a platform for the development of this publication. The authors would like to acknowledge the College of Health Sciences in the University of KwaZulu-Natal and eThekwini Municipality for all the valuable support.

Authors' contributions

SM conceptualized the study and wrote the manuscript. KH & MM contributed to the writing by critically reviewing the manuscript and making revisions. SM wrote the final draft manuscript and all the authors approved it.

Funding

The authors have not declared a specific grant for this research from any funding agency in the public, commercial or not-for-profit sectors.

Competing interests

None declared

Data availability statement

Data are available upon reasonable request. The datasets obtained and analysed during the current study are available from the corresponding author on reasonable request. Correspondence: 951030918@stu.ukzn.ac.za.

For peer review only - http://bmjopen.bmj.com/site/about/guidelines.xhtml

References

1. Shonhiwa AM, Ntshoe G, Essel V, Thomas J, McCarthy KJIJoID. A review of foodborne diseases outbreaks reported to the outbreak response unit, national institute for communicable diseases, South Africa, 2013–2017. 2019;79:73.

2. Linton RH, McSwane DZ. Foodborne Infections and Intoxications: Chapter 34. Food Safety Post-processing: Transportation, Supermarkets, and Restaurants: Elsevier Inc. Chapters; 2013.

3. Smith AM, Tau NP, Smouse SL, Allam M, Ismail A, Ramalwa NR, et al. Outbreak of Listeria monocytogenes in South Africa, 2017–2018: laboratory activities and experiences associated with whole-genome sequencing analysis of isolates. Foodborne pathogens and disease. 2019;16(7):524-30.

4. Organization WH. WHO estimates of the global burden of foodborne diseases: foodborne disease burden epidemiology reference group 2007-2015: World Health Organization; 2015.

5. da Vitória AG, Oliveira JdSC, de Almeida Pereira LC, de Faria CP, de São José JFB. Food safety knowledge, attitudes and practices of food handlers: A cross-sectional study in school kitchens in Espírito Santo, Brazil. BMC public health. 2021;21(1):1-10.

6. Organization WH. Estimating the burden of foodborne diseases: a practical handbook for countries: a guide for planning, implementing and reporting country-level burden of foodborne disease. 2021.

7. Ramalwa N, Page N, Smith A, Sekwadi P, Shonhiwa A, Ntshoe G, et al. Has foodborne disease outbreak notification and investigation changed since the listeriosis outbreak in South Africa? A review of foodborne disease outbreaks reported to the National Institute for Communicable Diseases, March 2018-August 2020.

8. Shonhiwa AM, Ntshoe G, Crisp N, Olowolagba AJ, Mbuthu V, Taylor MB, et al. Investigation of two suspected diarrhoeal-illness outbreaks in Northern Cape and KwaZulu-Natal provinces, South Africa, April–July 2013: The role of rotavirus. Southern African Journal of Infectious Diseases. 2020;35(1):8.

9. Shonhiwa AM, Ntshoe G, Essel V, Thomas J, McCarthy K. A review of foodborne diseases outbreaks reported to the outbreak response unit, national institute for communicable diseases, South Africa, 2013–2017. International Journal of Infectious Diseases. 2019;79:73.

10. Thomas J, Govender N, McCarthy KM, Erasmus LK, Doyle TJ, Allam M, et al. Outbreak of Listeriosis in South Africa Associated with Processed Meat. N Engl J Med. 2020;382(7):632-43.

For peer review only

For peer review only - http://bmjopen.bmj.com/site/about/guidelines.xhtml

11. Aalto-Araneda M, Lunden J, Markkula A, Hakola S, Korkeala H. Processing plant and machinery sanitation and hygiene practices associate with Listeria monocytogenes occurrence in ready-to-eat fish products. Food Microbiol. 2019;82:455-64.

12. Kaptchouang Tchatchouang CD, Fri J, De Santi M, Brandi G, Schiavano GF, Amagliani G, et al. Listeriosis Outbreak in South Africa: A Comparative Analysis with Previously Reported Cases Worldwide. Microorganisms. 2020;8(1).

13. Kuo C, Operario D. Caring for AIDS-orphaned children: An exploratory study of challenges faced by carers in KwaZulu-Natal, South Africa. Vulnerable children and youth studies. 2010;5(4):344-52.

14. KwaZulu-Natal DoHPo. District Health Plan 2015/2016 - EThekwini Health District, KwaZulu-Natal. 2015:120.

15. Heathcock R, McLauchlin J, Newton LH, Soltanpoor N, Coker R, Bignardi G, et al. Survey of food safety awareness among HIV positive individuals. AIDS care. 1998;10(2):237-41.

16. Todd EC, Greig JD, Bartleson CA, Michaels BS. Outbreaks where food workers have been implicated in the spread of foodborne disease. Part 2. Description of outbreaks by size, severity, and settings. Journal of food protection. 2007;70(8):1975-93.

17. Warshawsky DN. Urban Food Insecurity and the advent of food banking in southern Africa: African Food Security Urban Network (AFSUN); 2011.

18. da Cunha DT, de Rosso VV, Pereira MB, Stedefeldt E. The differences between observed and self-reported food safety practices: a study with food handlers using structural equation modeling. Food research international. 2019;125:108637.

19. Adane M, Teka B, Gismu Y, Halefom G, Ademe M. Food hygiene and safety measures among food handlers in street food shops and food establishments of Dessie town, Ethiopia: a community-based cross-sectional study. PloS one. 2018;13(5):e0196919.

20. Makhunga S, Mashamba-Thompson TP, Hlongwa M, Hlongwana KW. Evidence on Food Control System in Charitable Food Assistance System: A Systematic Scoping Review. 2018.

21. Donkor ES, Kayang BB, Quaye J, Akyeh ML. Application of the WHO keys of safer food to improve food handling practices of food vendors in a poor resource community in Ghana. Int J Environ Res Public Health. 2009;6.

22. Five keys to safer food manual. Geneva: WHO; 2006.

23. Bhat A. Snowball sampling: Definition, method, advantages and disadvantages. SNOWBALL SAMPLING: DEFINITION, METHOD, ADVANTAGES AND DISADVANTAGES; 2020.

24. McElrone M, Colby SE, Moret L, Kavanagh K, Spence M, Fouts HN, et al. Barriers and Facilitators to Food Security among Adult Burundian and Congolese Refugee Females Resettled in the US. Ecology of food and nutrition. 2019;58(3):247-64.

For peer review only

For peer review only - http://bmjopen.bmj.com/site/about/guidelines.xhtml

25. Fariba R, Gholamreza JK, Saharnaz N, Ehsan H, Masoud Y. Knowledge, attitude, and practice among food handlers of semi-industrial catering: a cross sectional study at one of the governmental organization in Tehran. Journal of Environmental Health Science and Engineering. 2018;16(2):249-56.

26. Kunadu AP-H, Ofosu DB, Aboagye E, Tano-Debrah K. Food safety knowledge, attitudes and self-reported practices of food handlers in institutional foodservice in Accra, Ghana. Food Control. 2016;69:324-30.

27. Nee SO, Sani NA. Assessment of knowledge, attitudes and practices (KAP) among food handlers at residential colleges and canteen regarding food safety. Sains Malaysiana. 2011;40(4):403-10.

28. Sani NA, Siow ON. Knowledge, attitudes and practices of food handlers on food safety in food service operations at the Universiti Kebangsaan Malaysia. Food control. 2014;37:210-7.

29. Abdi AM, Amano A, Abrahim A, Getahun M, Ababor S, Kumie A. Food hygiene practices and associated factors among food handlers working in food establishments in the Bole Sub City, Addis Ababa, Ethiopia. Risk Management and Healthcare Policy. 2020;13:1861.

30. Pollard CM, Mackintosh B, Campbell C, Kerr D, Begley A, Jancey J, et al. Charitable Food Systems' Capacity to Address Food Insecurity: An Australian Capital City Audit. Int J Environ Res Public Health. 2018;15(6).

31. De Boeck E, Jacxsens L, Goubert H, Uyttendaele M. Ensuring food safety in food donations: Case study of the Belgian donation/acceptation chain. Food Res Int. 2017;100(Pt 2):137-49.

32. De Pieri B, Tallarico T, Baglioni S, Soler U, Ricciuti E. European Policy for Food Security: The Surplus Food Redistribution Option. Foodsaving in Europe2017. p. 13-35.

33. Montesclaros J, Caballero-Anthony M, Schlundt J. Food Safety in ASEAN: Pitfalls of Complacency. 2018.

34. Regan Á, McConnon Á, Holah J. Food Hygiene and Food Workers: From Complacency to Compliance. Handbook of Hygiene Control in the Food Industry: Elsevier; 2016. p. 197-203.

35. Adzoyi PN, Honyenuga BQ. Exploring Knowledge, Attitudes and Practices towards Food Hygiene by Hospitality Students in Ghana. Journal of Food Sci and quality mgmt. 2014;26:2225-0557.

36. Sharif L, Obaidat MM, Al-Dalalah M-R. Food hygiene knowledge, attitudes and practices of the food handlers in the military hospitals. Food and Nutrition Sciences. 2013;4(03):245.

37. Al-Shabib NA, Mosilhey SH, Husain FM. Cross-sectional study on food safety knowledge, attitude and practices of male food handlers employed in restaurants of King Saud University, Saudi Arabia. Food Control. 2016;59:212-7.

For peer review only - http://bmjopen.bmj.com/site/about/guidelines.xhtml

38. Asmawi UMM, Norehan AA, Salikin K, Rosdi NAS, Munir NATA, Basri NBM. An assessment of knowledge, attitudes and practices in food safety among food handlers engaged in food courts. Current Research in Nutrition and Food Science Journal. 2018;6(2):346-53.

39. Bou-Mitri C, Mahmoud D, El Gerges N, Abou Jaoude M. Food safety knowledge, attitudes and practices of food handlers in lebanese hospitals: A cross-sectional study. Food control. 2018;94:78-84.

40. El-Nemr I, Mushtaha M, Irungu P, Asim H, Tang P, Hasan M, et al. Assessment of food safety knowledge, self-reported practices, and microbiological hand hygiene levels of produce handlers in Qatar. Journal of food protection. 2019;82(4):561-9.

For peer review only

For peer review only - http://bmjopen.bmj.com/site/about/guidelines.xhtml

BMJ Open

Cross-sectional study on food handlers' knowledge, attitudes, and self-reported practices regarding safe food handling in charitable food assistance programs in the eThekwini District, South Africa

Journal:	*BMJ Open*
Manuscript ID	bmjopen-2022-065357.R1
Article Type:	Original research
Date Submitted by the Author:	10-Jan-2023
Complete List of Authors:	Makhunga, Sizwe; University of KwaZulu-Natal College of Health Sciences, Public Health<br>Macherera, Margaret; Lupane State University, Department of Crop and Soil Sciences<br>Hlongwana, Khumbulani; University of KwaZulu-Natal, Department of Public Health Medicine; University of KwaZulu-Natal
**Primary Subject Heading**:	Public health
Secondary Subject Heading:	Public health, Health policy, Nutrition and metabolism
Keywords:	Nutritional support < GASTROENTEROLOGY, Health & safety < HEALTH SERVICES ADMINISTRATION & MANAGEMENT, Health policy < HEALTH SERVICES ADMINISTRATION & MANAGEMENT, Public health < INFECTIOUS DISEASES

SCHOLARONE™
Manuscripts

I, the Submitting Author has the right to grant and does grant on behalf of all authors of the Work (as defined in the below author licence), an exclusive licence and/or a non-exclusive licence for contributions from authors who are: i) UK Crown employees; ii) where BMJ has agreed a CC-BY licence shall apply, and/or iii) in accordance with the terms applicable for US Federal Government officers or employees acting as part of their official duties; on a worldwide, perpetual, irrevocable, royalty-free basis to BMJ Publishing Group Ltd ("BMJ") its licensees and where the relevant Journal is co-owned by BMJ to the co-owners of the Journal, to publish the Work in this journal and any other BMJ products and to exploit all rights, as set out in our licence.

The Submitting Author accepts and understands that any supply made under these terms is made by BMJ to the Submitting Author unless you are acting as an employee on behalf of your employer or a postgraduate student of an affiliated institution which is paying any applicable article publishing charge ("APC") for Open Access articles. Where the Submitting Author wishes to make the Work available on an Open Access basis (and intends to pay the relevant APC), the terms of reuse of such Open Access shall be governed by a Creative Commons licence – details of these licences and which Creative Commons licence will apply to this Work are set out in our licence referred to above.

Other than as permitted in any relevant BMJ Author's Self Archiving Policies, I confirm this Work has not been accepted for publication elsewhere, is not being considered for publication elsewhere and does not duplicate material already published. I confirm all authors consent to publication of this Work and authorise the granting of this licence.

For peer review only

Cross-sectional study on food handlers' knowledge, attitudes, and self-reported practices regarding safe food handling in charitable food assistance programs in the eThekwini District, South Africa

Sizwe Makhunga[1], Margaret Macherera[2], and Khumbulani Hlongwana[1,3]

[1]Department of Public Health Medicine, School of Nursing and Public Health, University of KwaZulu-Natal, Durban, South Africa

[2]Department of Crop and Soil Sciences, Faculty of Agricultural Sciences, Lupane State University, Lupane, Zimbabwe

[3]Cancer and Infectious Diseases Epidemiology Research Unit (CIDERU), School of Nursing and Public Health, University of KwaZulu-Natal, Durban, South Africa

Corresponding Author: Sizwe Makhunga (Ph.D. Candidate)

Address: 2nd Floor George Campbell Building, Department of Public Health Medicine, School of Nursing and Public Health, University of KwaZulu-Natal, Durban, 4001, South Africa

Email address: makhungas@gmail.com/ 951030918@stu.ukzn.ac.za

Tel: +27730623901/ +27313229852

Email addresses of authors

Khumbulani Hlongwana (Hlongwanak@ukzn.ac.za)

Margaret Macherera (mmacherera@gmail.com)

For peer review only

Abstract

Objective: This study aimed to assess food handlers' knowledge, attitudes, and self-reported practices towards the safer donated foods.

Design This cross-sectional study was conducted from January to May 2021.

Setting: This study was conducted in the eThekwini District, South Africa.

Participants: A convenience sampling-based cross-sectional survey of food handlers (n = 252) served as the study's main source of data. A total of eight study sites across five municipal planning regions (MPRs) of eThekwini District were visited. The principal investigator administered the validated structured standardised questionnaire, using participant's language of choice, which was either English or isiZulu. All the questions in the questionnaire were asked in exactly the same manner, following the same flow, in order to avoid bias and ensure consistency. Furthermore, the close-ended nature of questions in the questionnaire mitigated the risks of question manipulation. The questionnaire was adapted from the World Health Organization's (WHO) five keys to a safer food manual.

Primary and secondary outcome measures: The primary outcome was the knowledge, attitudes, and self-reported practices towards the safer donated foods. The secondary outcomes were the sanitary conditions of infrastructure and facilities, food handlers' observed food handling behaviour, and nutritional information/labeling.

Results: The results show that the food handlers had good knowledge, positive attitude, and acceptable behaviours towards safe food handling and general hygiene, with mean scores (SD) of 62,8 (14,6), 92,5 (14,1), and 80,4 (13,3), respectively. Significant correlations were found between knowledge and attitudes (p = 0,001), knowledge and practices (p<0,001), and attitudes and practices (p=0,02). However, the correlations between knowledge versus attitude and attitude versus practice were poor (spearman's rho <0,3), and the association between knowledge versus practice was moderate (0,3 – 0,7). The majority of food handlers (92,5%) did not understand the value of thorough cooking and temperature control. About 53% of respondents acknowledged to never adequately reheating cooked meals, 5% did not see the significance of preventing cross-contamination, and 5% were unsure.

Conclusions: Despite the relatively positive KAP levels of the food handlers, safe food handling and hygiene practices, such as thorough cooking and temperature control, properly reheating cooked meals, and taking precautions to prevent cross-contamination, require some emphasis.

For peer review only - http://bmjopen.bmj.com/site/about/guidelines.xhtml

Keywords: Knowledge, Attitudes, Self-reported practices, Food handlers, Charitable food assistance programs

Strengths and Limitations of this Study

- The sample size of the study was not too large. However, the diverse CFAPs from which the sample was drawn offer unique strength to the study.
- The study was prone to social desirability bias, given that most data emanated from self-reporting.
- Even though the sampling was thorough and included every CFAP in the database, it's possible that some participants were left out.
- The walkthrough survey, which was used to collect observational data in addition to survey data in order to supplement survey results and lessen the impacts of social desirability bias, was not included in the study.

Introduction

Foodborne diseases (FBDs) resulting from contaminated food continue to pose significant public health challenges worldwide, particularly in low and middle-income countries (LMICs), where socio-economic inequities are pervasive (1). FBDs are a diverse group of illnesses caused by eating foods contaminated with infectious or poisonous microbes and/or chemicals (2). Pathogenic microorganisms, such as bacteria, viruses, parasites, fungus, and chemicals, are the primary causes of FBDs (3). In the clinical environment, FBDs are most exhibited as gastrointestinal symptoms. Other symptoms, such as neurological, immunological, and gynaecological issues, may also occur (2).

While unsafe food is a global health hazard, infants, small children, the elderly, pregnant women, and persons with underlying illnesses are most susceptible, putting them at greater risk for severe FBD (4-6). FBDs are disproportionately distributed globally, with Africa having the highest rate of FBD per capita globally. Infections associated with diarrhoeal diseases account for a significant proportion of the cases (4). The high incidence of FBD is due to the usage of unfit water for cleaning and processing food, inefficient food production, poor food handling, insufficient food-storage infrastructure, and regulatory policies that are either ineffective or ineffectively enforced (7-9). The listeriosis outbreak of 2017 to 2018 in South Africa was a

For peer review only - http://bmjopen.bmj.com/site/about/guidelines.xhtml

reminder of the potentially catastrophic consequences of the FBD outbreak, underscoring the need to improve food safety controls and interventions (1) and (3). Nine hundred and thirty-seven cases were found, 465 (50%) of which were linked to pregnancy, and 406 (87%) of the pregnancy-related cases occurred in neonates. Two hundred and twenty-nine (24%) of the 937 cases involved individuals aged 15 to 49, excluding those who were pregnant. Among the patients whose HIV status was known, 38% of those with pregnancy-related cases (77 of 204) and 46% of the remaining patients (97 of 211) were infected with the listeria virus. One hundred and ninety-three (27%) of the 728 patients with a known outcome died (10-12).

In KwaZulu-Natal (KZN), the health risks associated with unsafe food handling are prevalent, owing to the high incidence of TB, HIV, and AIDS in the province (13, 14). Research has shown that a seemingly minor foodborne infection can be lethal in immunocompromised people (15). According to the National Institute for Communicable Diseases (NICD) Review Report on FBD outbreaks, the KwaZulu-Natal province had the most (5 cases per 100 000 population) FBD outbreaks between March 2018 and March 2020, outnumbering all nine provinces in South Africa. (7). Research has shown that malnutrition and diarrhoea caused by consuming contaminated food are the leading causes of infant mortality in hygiene-challenged settings like the eThekwini District (16).

Fifty-four percent (54%) of the unemployed in the KZN province live in the eThekwini district, thereby making the district vulnerable to health conditions exacerbated by poverty. For example, 31% of the population in the eThekwini district is poor, putting the district at increased risk of food insecurity (14). As a result, there is a strong reliance on government services and charitable food assistance programs (CFAPs). For the purpose of this study, CFAPs are described as food establishments that gather and distribute surplus food to people who are food-insecure (10, 11). These organizations include food banks, food rescue organizations, pantries, community soup kitchens, child and youth care centers, and emergency shelters (10, 12). Despite several CFAPs operating in the eThekwini district for decades, there is a lack of data about the status of their safety and hygiene practices. However, a scoping review of studies from high-income countries (HICs) by Makhunga *et al*. (17) revealed that the majority of food handlers employed by CFAPs are untrained volunteers who work on a volunteer basis.

For peer review only - http://bmjopen.bmj.com/site/about/guidelines.xhtml

Food handlers' knowledge, attitudes, and practices towards safe food handling are essential in improving food safety and reducing inadvertent foodborne illnesses (5, 18). Food contamination caused by poor personal hygiene, particularly inefficient hand washing, has been identified as an essential risk factor for food poisoning (19). Unlike in the conventional food supply chain, there is a lack of data on hygiene practices regarding food handling in CFAPs (20, 21). Thus, this study investigated food handlers' knowledge, attitudes, and self-reported practices of food handling on five keys to safer food, based on the World Health Organization's (WHO) five keys to a safer food manual (22, 23) in the eThekwini District's CFAPs in South Africa. The findings of this research can be utilized to help develop strategies for enhancing food safety in CFAPs and, in turn, protecting vulnerable populations from foodborne infections.

Materials and methods

The study area

This study was conducted in eThekwini District, South Africa, between January and May 2021. EThekwini District is one of the eleven Districts in KwaZulu-Natal (KZN), South Africa. It comprises 103 urban, rural, and peri-rural wards, which spread across eight sub-districts: South Central, South-West, Umlazi and Engonyameni, Lower South, North Central, Greater Inanda /Tongaat Sub-District, Inner-West, and Outer West. It covers approximately 2,556 km^2 area stretching from the Umkomaas in the south, Tongaat in the north, and Cato Ridge in the west, including a few tribal areas in Umbumbulu, and Ndwedwe. It has an estimated population of 3,702,231 (2016 estimate) and a population density of 1,448.5 people per km^2, with a sizeable population settling in the South Region (41%), followed by the North Region (32%) and the West Region (27%) (14). Despite being highly urbanized and densely populated, pockets of rural communities exist on the west, south, and north outskirts, impacting equitable access to essential services (14).

Research design and sampling

A convenience sampling-based cross-sectional survey of food handlers (n = 252) served as the study's main source of data. A total of eight study settings (South Central, South-West, Umlazi and Engonyameni, Lower South, North Central, Greater Inanda /Tongaat Sub-District, Inner-West, and Outer West) across five (North, Central, South, Inner, and Outer West) municipal planning regions (MPRs) of eThekwini District were visited and participated in the study.

For peer review only - http://bmjopen.bmj.com/site/about/guidelines.xhtml

Multi-staged sampling

Stage one – Identification of CFAPs

As a first step in creating a database of CFAPs, we checked the website of the National Department of Social Development (NDSD). We also approached the FoodForward SA Durban to obtain the list of CFAPs in their database because the process was not exhaustive. The two databases were combined to create a single comprehensive database that had 196 CFAPs. The study included all 196 CFAPs, making it a population sampling.

Stage two – Identification of participants

It was decided that a sample size calculation of 192 was sufficient. We could only get 196 CFAPs, which meant that one participant per CFAP would yield the desired outcome. However, in CFAPs with a high staff volume, we allowed for a maximum of three participants. The convenience sampling approach meant that everyone who met the criteria, was accessible, willing to participate, and available was included.

Data collection

A researcher administered the structured standard questionnaire face-to-face to all sampled and consenting food handlers (n=252) in the selected CFAPs (n=196). A minimum of one and a maximum of three food handlers were recruited in each of the 196 participating CFAPs. The CFAPs that were chosen were visited during their usual business hours. Before participating in the study, each subject signed an informed consent form. The inclusion criteria were all persons involved in food handling. The principal investigator administered the validated structured standardised questionnaire, using participant's language of choice, which was either English or isiZulu.

All the questions in the questionnaire were asked in exactly the same manner, following the same flow, in order to avoid bias and ensure consistency. Furthermore, the close-ended nature of questions in the questionnaire mitigated the risks of question manipulation. The questionnaire was adapted from the World Health Organization's (WHO) five keys to a safer food manual. We adapted the questionnaire from the World Health Organization's (WHO) five keys to a safer food manual (24-26). The questionnaire was pilot tested in ten CFAPs at the Umsunduzi Municipality in Pietermaritzburg, South Africa. Cronbach's alpha coefficient (α) of internal consistency (27) was used to estimate the reliability of the questionnaire, which was

For peer review only - http://bmjopen.bmj.com/site/about/guidelines.xhtml

found to be acceptable at 0.76. Therefore, since there were no issues with the questionnaire, no changes were required.

Research instrument

The questionnaire was adapted from the World Health Organization's 'Five keys to safer food' guideline for food handlers (23). We used the manual of the five keys available on the WHO site, first released in a poster in 2001. This handbook includes two sections: section one provides background information for the manual, and section two stipulates five keys to safer food. The participants' food safety knowledge, attitude, and self-reported behavior were assessed using three forms found at the end of section two of the handbook. The questionnaire was structured into six distinctive parts to collect information (i) demographic characteristics, (ii) background characteristics of the CFAPs, (iii) food hygiene & safety practices and general requirements, (iv) knowledge, (v) attitude, and (vi) self-reported practices. However, this paper focuses on four parts, excluding parts two and three. We also used a checklist to gather data during walkthrough surveys and observations of practices. However, the findings of the walkthrough survey did not form part of this study.

Statistical analysis

All statistical analyses were performed using StataCorp. 2021. *Stata Statistical Software: Release 17*. College Station, TX: StataCorp LLC. Chi-square test was used to determine the relationship between the sociodemographic characteristics of the food handlers and their knowledge, attitudes, and self-reported practices level. We used descriptive statistics to summarize the CFAPs and food-services staff's general characteristics and describe their food hygiene and safety knowledge, attitudes, and self-reported practices. By categorizing answers for each section as dichotomous variables, statistical analysis of the relationship between questionnaire answers and demographic characteristics of respondents was performed: knowledge was classified and recorded as correct vs. incorrect, attitudes as agreement vs. disagreement/uncertain, and practices as safe, when an answer was always, vs. unsafe, when an answer was often or never.

Only the questions with a proportion of accurate answers of 95 percent or less were reported. The level of knowledge of the food handlers was determined through the following criteria, namely: each correct response received one point, while each incorrect answer received zero points. The score allocation for attitude was two points for agreeing, one point for not sure, and

For peer review only - http://bmjopen.bmj.com/site/about/guidelines.xhtml

a zero for disagree. Based on the response to 11 knowledge questions, the scores could vary from 0 to 11. Three levels were considered for this: scores between 0 to 4 were classified as poor, 5 to 7 as moderate, and 8 to 11 as good. Total scores could differ for ten attitude questions, and 0 to 20 classifications were allocated: 0 to 6 being negative, 7 to 13 being neutral, and 14 to 20 being positive attitude levels. For ten practice questions, participants could choose from options 1 to 5, where one referred to never and 5 to always. Scores were allocated as follows: always (4), mostly (3), sometimes (2), rarely (1), and never (0). Total scores could differ for ten practice questions, and 0 to 20 classifications were allocated: 0 to 6 being poor, 7 to 13 being moderate, and 14 to 20 being acceptable practice levels.

Patient and public involvement

Patients and/or the public were not involved in the design, conduct, reporting, or dissemination plans of this study.

Results

Distribution of study participants among sub-districts

A total of 252 food handlers participated, most coming from the Greater Inanda/Tongaat Sub-District and Lower South (**Table 1**).

Table 1: Distribution of participating CFAPs (n = 196) and study participants (n = 252)

Location	CFAPs (n=196)	Food handlers (n=252)
South Central	28	40
South-West	12	18
Umlazi and Engonyameni	14	20
Lower South	42	50
North Central	36	40
Greater Inanda /Tongaat Sub-District	54	70
Inner West	4	8
Outer West	6	6

Sociodemographic characteristics of the food handlers

Ninety-four percent (n = 237/252) of the food handlers were females, with nearly half (49,2%) being over 50 years (**Table 2**). Less than half (40,5%) of food handlers had a Grade 12 (Matric) certification, while most (59,1%) had a post-matric qualification. The majority (72,2%) of

For peer review only - http://bmjopen.bmj.com/site/about/guidelines.xhtml

For peer review only

participants had more than ten years of service experience, and 60,7% related to the food service industry. All food handlers (100%) had direct contact with food during receiving, checking, and storage. As high as 80% of them were involved in food preparation and processing/cooking. The majority (88,1%) of participants reported their primary role as serving, packaging & distribution, with only 14,7% washing and cleaning the food facility. Only 12,3% of food handlers had attended at least one official training session on food hygiene elements within five years preceding this study, while the majority (87,7%) reported to have had no training in food safety (**Table 2**).

Table 2: Demographic characteristics of food handlers (n = 252)

Socio demographic characteristics	Frequency (n)	Percentage (%)
**Gender**		
Male	15	6,0
Female	237	94,0
**Age group (years)**		
20 – 29	14	5,5
30 – 39	36	14,3
40 – 49	78	31,0
50 +	124	49,2
**Race**		
Black	144	57,1
White	19	7,5
Indian	69	27,4
Coloured	20	7,9
**Marital Status**		
Single	106	42,1
Married	107	42,5
Divorced	29	11,5
Widowed	10	4,0
**Number of dependents**		
None	24	9,5
<2	67	26,6
2-5	124	49,2
> 5	37	14,7
**Place of residence**		
Formal settlement	181	71,8
Informal settlement	3	1,2
Shelter	68	27,0
**Education level**		
Primary	1	0,4
Secondary	102	40,5
Tertiary	149	59,1
**Overall work experience (years)**		

For peer review only

3-5	18	7,1
6-10	52	20,6
> 10	182	72,2
**Experience in food service (years)**		
< 1	6	2,4
1-3	23	9,1
4-6	53	21,0
7-10	17	6,7
> 10	153	60,7
**Main Job responsibility**		
Food receiving, checking & storage	252	100,0
Food preparation, process/cooking	202	80,2
Food serving/packaging/distribution	222	88,1
Washing and cleaning	37	14,7
Other	0	0,0
**Attended safe food handling training**		
Yes	31	12,3
No	221	87,7
If yes, the date (years)		
≤ 2	17	6,7
3-4	14	5,6
N/A	221	87,7

Knowledge

All food handlers (100%) knew it was essential to wash their hands before handling food (**Table 3**). About 85% of food handlers were aware of the dangers of food cross-contamination and consequently reported that they had taken steps to avoid it. However, 17% of food handlers were unaware that wiping cloths can spread bacteria, primarily when used filthy. Most food handlers (71%) were enthusiastic about safe food handling. However, 53% admitted never properly reheating cooked meals when asked about their food handling methods. Most (92,5%) of the food handlers did not appreciate the importance of thorough cooking and temperature control. Although almost all food handlers (99,6%) reported that they wash fruits and vegetables, the majority (90,7%) thought one could tell water safety by simply looking at it. The overall results demonstrated that about 41% of food handlers in CFAPs have poor knowledge of safe food handling and general hygiene.

Table 3: Food handlers' knowledge of safe food handling in charitable food assistance programs (n = 252)

Statement/Question	True No (%)	False No (%)
**1.** It is essential to wash hands before handling food	252 (100)	0 (0)

For peer review only - http://bmjopen.bmj.com/site/about/guidelines.xhtml

**2.**	Wiping cloths can spread microorganisms	208 (82,5)	44 (17,5)
**3.**	The same cutting board can be used for raw and cooked foods, provided it looks clean	39 (15,5)	213 (84,5)
**4.**	Raw food needs to be stored separately from cooked food	216 (85,7)	36 (14,3)
**5.**	Cooked foods do not need to be thoroughly reheated	186 (73,8)	66 (26,2)
**6.**	Proper cooking includes meat cooked to 40 °C	233 (92,5)	19 (7,5)
**7.**	Cooked meat can be left at room temperature overnight to cool before refrigerating	62 (24,6)	190 (75,4)
**8.**	Cooked food should be kept very hot before serving	148 (58,7)	104 (41,3)
**9.**	Refrigerating food only slows bacterial growth	154 (61,1)	98 (38,9)
**10.**	Safe water can be identified by the way it looks	229 (90,9)	23 (9,1)
**11.**	Wash fruit and vegetables	251 (99,6)	1 (0,4)

Attitude

All the food handlers (100%) agreed that it is crucial to observe general hygiene, and 89,3% believed using different knives and cutting boards for raw and cooked foods is worth the extra effort. However, 5,2% did not show appreciation of the importance of guarding against cross-contamination, and 5,5% were uncertain. Almost all food handlers (96,8%) reported ensuring thorough cooking and observed temperature controls. They were also aware of the consequences of improperly thawing food. Furthermore, 75% of food handlers believe that leaving prepared food at room temperature for more than two hours without refrigeration is dangerous (**Table 4**). With a score of 96.8%, the food handlers in CFAPs demonstrated a positive attitude toward safe food handling and general hygiene.

Table 4: Attitudes of food handlers towards safe food handling in charitable food assistance programs (n = 252)

**Statement/Question**		**Agree No (%)**	**Unsure No (%)**	**Disagree No (%)**
**1.**	Frequent handwashing during food preparation is worth the extra time	252 (100)	0 (0)	0 (0)
**2.**	Keeping kitchen surfaces clean reduces the risk of illness	252 (100)	0 (0)	0 (0)
**3.**	Keeping raw and cooked food separate helps to prevent illness	225 (89,3)	27 (10,7)	0 (0)
**4.**	Using different knives and cutting boards for raw and cooked foods is worth the extra effort	225 (89,3)	14 (5,5)	13 (5,2)
**5.**	Meat thermometers help ensure food is cooked thoroughly	196 (77,8)	29 (11,5)	27 (10,7)
**6.**	Soups and stews should always be boiled to ensure the safety	244 (96,8)	0 (0)	8 (3,2)
**7.**	Thawing food in a cool place is safer	244 (96,8)	8 (3,2)	0 (0)

For peer review only - http://bmjopen.bmj.com/site/about/guidelines.xhtml

**8.** I think it is unsafe to leave cooked food out of the refrigerator for more than two hours	189 (75,0)	28 (11,1)	35 (13,9)
**9.** Inspecting food for freshness and wholesomeness is valuable	252 (100)	0 (0)	0 (0)
**10.** I think it is important to throw away foods that have reached their expiry date	252 (100)	0 (0)	0 (0)

Self-reported practices

All food handlers (100%) stated that they always maintain a high level of general hygiene (**Table 5**). Nearly two-thirds (65,9%) of the participants used separate surfaces for cooked and ready-to-eat food. However, 84,9% of the participants reported segregating raw and cooked food during storage. Food handlers reported observed temperature control in 68,3 percent and 55,6 percent of cases, respectively. Less than half (43,7%) of the participants said it was necessary to reheat cooked food until it was boiling. All food handlers (100%) said they inspect and discard food that has passed its expiration date. They also asserted that they wash fruits and vegetables before consuming them with clean water. The food handlers in CFAPs reported acceptable general hygiene and safe food handling practices with a score of 100%.

Table 5: Self-reported practices of food handlers in charitable food assistance programs (n = 252)

Statement/Question	Always No (%)	Mostly No (%)	Sometimes No (%)	Rarely No (%)	Never No (%)
**1.** I wash my hands before and during food preparation	252 (100)	0 (0)	0 (0)	0 (0)	0 (0)
**2.** I clean surfaces and equipment used for food preparation before re-using on other food	252 (100)	0 (0)	0 (0)	0 (0)	0 (0)
**3.** I use separate utensils and cutting boards when preparing raw and cooked food	166 (65,9)	59 (23,1)	0 (0)	0 (0)	27 (11)
**4.** I separate raw and cooked food during storage	214 (84,9)	9 (3,6)	0 (0)	0 (0)	29 (12)
**5.** I check that meats are cooked thoroughly by ensuring that the juices are clear or by using a thermometer	217 (86,0)	0 (0)	0 (0)	0 (0)	35 (14)
**6.** I reheat cooked food until it is piping hot throughout	110 (43,7)	6 (2,4)	0 (0)	0 (0)	134 (53)
**7.** I thaw frozen food in the refrigerator or other cool place	140 (55,6)	7 (28)	23 (9,1)	0 (0)	82 (33)
**8.** After I have cooked a meal, I store any leftovers in a cool place within two hours	172 (68,3)	2 (0,8)	57 (22,6)	0 (0)	21 (8)

For peer review only - http://bmjopen.bmj.com/site/about/guidelines.xhtml

**9.** I check and throw away food beyond its expiry date	252 (100)	0 (0)	0 (0)	0 (0)	0 (0)
**10.** I wash fruit and vegetables with safe water before eating them	252 (100)	0 (0)	0 (0)	0 (0)	0 (0)

1.1 Association among the food safety knowledge, attitudes, and practices of food handlers

Significant correlations (**Table 6**) were found between knowledge and attitudes (p = 0,001), knowledge and practices (p<0,001), and attitudes and practices (p=0,02). However, the correlations between knowledge vs. attitude and attitude vs. practice were poor (spearman's rho < 0,3), and the association between knowledge vs. practice was moderate (0,3 – 0,7).

Table 6: *Association among food safety knowledge, attitudes, and practices*

Level	Spearman's rho	Significance
Knowledge vs. Attitude	0,25	0,001
Knowledge vs. Practice	0,43	<0,001
Attitude vs. Practice	0,15	0,02

Discussion

This study showed that food handlers in CFAPs had relatively good knowledge, attitudes, and practices. This is despite most food handlers (87,7%) lacking training in food safety, with only 12,3% have attended at least one official training course on food safety and general hygiene within five years of the study. Staff retention appeared to be somewhat high, given that 72,2% of food handlers had more than ten years of service experience, 60,7% of which were in the food service industry. All food handlers (100%) understood the importance of washing their hands before handling food. Although 85% of food handlers knew the risks of food cross-contamination, 17% were unaware that wiping cloths can spread bacteria, mainly when used filthy. Almost all (92,5%) food handlers participating in this study did not realize the importance of thorough cooking and temperature control, as evidenced by 53% of food handlers who admitted to never properly reheating cooked meals. Although almost all food handlers (99,6%) reported that they wash fruits and vegetables, the majority (90,7%) thought one could tell water safety by simply looking at it.

For peer review only

According to this study, food handlers in the CFAPs recounted good knowledge, positive attitudes, and good self-reported practices related to safe food handling. The findings are consistent with other studies conducted in the conventional food supply chain in LMICs settings (28-32). In their recent study, Fariba et al. (22) found that food handlers were good at separating raw and cooked materials and using clean water and cloth. Their findings are consistent with ours, revealing that most (85%) food handlers in CFAPs knew the risks of food cross-contamination. This is likely due to extensive food handling experience accrued over a long time, as most food handlers had over ten years of experience, especially in the foodservice industry. When interviewed, some food handlers asserted that they had worked for big corporate restaurants before joining the CFAPs as food service staff. Their knowledge and experience can be enhanced with current and formal routine food safety training. Local authorities can support CFAPs by rendering services such as regular inspections, food sampling, certification/permitting, seizure and disposal of expired foodstuffs, and health and hygiene education and training, as seen in high-income countries (33-35).

The current study's findings support prior research (27-30), revealing that most food handlers in CFAPs are reckless with donated food. This is because while volunteering, no training in proper food handling is provided to food handlers. However, it was discovered that most food handlers (72,2%) had more than ten years of experience and that 60,7% of the workers had spent most of their working lives in the food service industry. While this may appear to be good because workers have gained a lot of experience over time, several studies have warned against complacency as the job becomes dull and workers lose interest in their profession. Most food handlers become casual with safe food handling at this point, putting the food in danger of contamination (36-38). Significant correlations were found between knowledge and attitudes ($p = 0,001$), knowledge and practices ($p<0,001$), and attitudes and practices ($p=0,02$). However, the correlations between knowledge versus attitude and attitude versus practice were poor (spearman's rho $< 0,3$), and the association between knowledge versus practice was moderate ($0,3 - 0,7$). This means that it is impossible to say whether respondents' food safety knowledge will influence their attitude, though it may influence their practices around food safety. The findings also show that food handlers' attitudes toward food safety do not accurately reflect their actual food safety practices.

On the other hand, some CFAPs food handlers claimed a lack of basic infrastructure and resources, such as limited workstations, poor food preparation/processing equipment, and

For peer review only - http://bmjopen.bmj.com/site/about/guidelines.xhtml

inadequate storage facilities, to name but a few. The walkthrough inspections and observations by the researcher also corroborated the reports by some CFAP food handlers. These challenges make it difficult for CFAP's food handlers to put their knowledge and competence acquired over the years to optimal use. Nevertheless, CFAP food handlers' understanding of proper reheating of cooked food, safe water by appearance, food holding temperature, and the danger zone remained weak. These findings were consistent with the conclusions made by Sharif et al. (39), asserting that despite good knowledge levels among food handlers, they had insufficient knowledge on thawing, thorough cooking, reheating, and safe temperature. Other studies have shown that this could pose potential risks or cause food poisoning in consumers (40-43).

The study's key weakness is the lack of similar studies published in South Africa. The sample size of the study was not too large. However, the diverse CFAPs from which the sample was drawn offer unique strength to the study. However, our study was prone to social desirability bias, given that most data emanated from self-reporting. However, the infusion of a walkthrough as part of the data collection technique to complement the survey questionnaire, alongside the comprehensive nature of the consent process, served as essential mitigation. Nonetheless, the current findings can be used to support the promotion of food handler training, particularly in CFAPs. Based on our results, we suggest that future research investigate the perceived barriers and facilitators to food safety and general hygiene in CFAPs.

Conclusion

Despite the relatively positive KAP levels of the food handlers, safe food handling and hygiene practices, such as thorough cooking and temperature control, properly reheating cooked meals, and taking precautions to prevent cross-contamination, require some emphasis. To reduce the risk of foodborne illness, the CFAPs' food service workers should get regular health and hygiene instruction as well as food safety training and relevant manuals.

Statements

Acknowledgments

IREX funding through the UASP alumni small grants program is acknowledged for providing a platform for developing this publication. The authors would like to recognize the College of Health Sciences at the University of KwaZulu-Natal and eThekwini Municipality for all the valuable support.

For peer review only - http://bmjopen.bmj.com/site/about/guidelines.xhtml

Authors' contributions

SM conceptualized the study and wrote the manuscript. KH & MM contributed to the writing by critically reviewing the manuscript and making revisions. SM wrote the final draft manuscript, and all the authors approved it.

Funding

The authors have not declared a specific grant for this research from any funding agency in public, commercial or not-for-profit sectors.

Competing interests

None declared

Ethics approval

The ethical clearance (BREC/00000821/2019) was obtained from the University of KwaZulu-Natal Biomedical Research Ethics Committee (BREC). The eThekwini District approved the study, and all participants consented to participate in the study.

Data availability statement

Extra data can be accessed via the Dryad data repository at http://datadryad.org/ with the doi: 10.5061/dryad.g1jwstqvd

References

1. Shonhiwa AM, Ntshoe G, Essel V, Thomas J, McCarthy KJIJoID. A review of foodborne diseases outbreaks reported to the outbreak response unit, national institute for communicable diseases, South Africa, 2013–2017. 2019;79:73.

2. Linton RH, McSwane DZ. Foodborne Infections and Intoxications: Chapter 34. Food Safety Post-processing: Transportation, Supermarkets, and Restaurants: Elsevier Inc. Chapters; 2013.

3. Smith AM, Tau NP, Smouse SL, Allam M, Ismail A, Ramalwa NR, et al. Outbreak of Listeria monocytogenes in South Africa, 2017–2018: laboratory activities and experiences associated with whole-genome sequencing analysis of isolates. Foodborne pathogens and disease. 2019;16(7):524-30.

For peer review only - http://bmjopen.bmj.com/site/about/guidelines.xhtml

4. Organization WH. WHO estimates of the global burden of foodborne diseases: foodborne disease burden epidemiology reference group 2007-2015: World Health Organization; 2015.

5. da Vitória AG, Oliveira JdSC, de Almeida Pereira LC, de Faria CP, de São José JFB. Food safety knowledge, attitudes and practices of food handlers: A cross-sectional study in school kitchens in Espírito Santo, Brazil. BMC public health. 2021;21(1):1-10.

6. Organization WH. Estimating the burden of foodborne diseases: a practical handbook for countries: a guide for planning, implementing and reporting country-level burden of foodborne disease. 2021.

7. Ramalwa N, Page N, Smith A, Sekwadi P, Shonhiwa A, Ntshoe G, et al. Has foodborne disease outbreak notification and investigation changed since the listeriosis outbreak in South Africa? A review of foodborne disease outbreaks reported to the National Institute for Communicable Diseases, March 2018-August 2020.

8. Shonhiwa AM, Ntshoe G, Crisp N, Olowolagba AJ, Mbuthu V, Taylor MB, et al. Investigation of two suspected diarrhoeal-illness outbreaks in Northern Cape and KwaZulu-Natal provinces, South Africa, April–July 2013: The role of rotavirus. Southern African Journal of Infectious Diseases. 2020;35(1):8.

9. Shonhiwa AM, Ntshoe G, Essel V, Thomas J, McCarthy K. A review of foodborne diseases outbreaks reported to the outbreak response unit, national institute for communicable diseases, South Africa, 2013–2017. International Journal of Infectious Diseases. 2019;79:73.

10. Thomas J, Govender N, McCarthy KM, Erasmus LK, Doyle TJ, Allam M, et al. Outbreak of Listeriosis in South Africa Associated with Processed Meat. N Engl J Med. 2020;382(7):632-43.

11. Aalto-Araneda M, Lunden J, Markkula A, Hakola S, Korkeala H. Processing plant and machinery sanitation and hygiene practices associate with Listeria monocytogenes occurrence in ready-to-eat fish products. Food Microbiol. 2019;82:455-64.

12. Kaptchouang Tchatchouang CD, Fri J, De Santi M, Brandi G, Schiavano GF, Amagliani G, et al. Listeriosis Outbreak in South Africa: A Comparative Analysis with Previously Reported Cases Worldwide. Microorganisms. 2020;8(1).

13. Kuo C, Operario D. Caring for AIDS-orphaned children: An exploratory study of challenges faced by carers in KwaZulu-Natal, South Africa. Vulnerable children and youth studies. 2010;5(4):344-52.

14. KwaZulu-Natal DoHPo. District Health Plan 2015/2016 - EThekwini Health District, KwaZulu-Natal. 2015:120.

15. Heathcock R, McLauchlin J, Newton LH, Soltanpoor N, Coker R, Bignardi G, et al. Survey of food safety awareness among HIV positive individuals. AIDS care. 1998;10(2):237-41.

For peer review only

16. Todd EC, Greig JD, Bartleson CA, Michaels BS. Outbreaks where food workers have been implicated in the spread of foodborne disease. Part 2. Description of outbreaks by size, severity, and settings. Journal of food protection. 2007;70(8):1975-93.

17. Makhunga S, Mashamba-Thompson T, Hlongwa M, Hlongwana K. Evidence on food control in charitable food assistance programs: a systematic scoping review. Syst Rev. 2019;8(1):240.

18. da Cunha DT, de Rosso VV, Pereira MB, Stedefeldt E. The differences between observed and self-reported food safety practices: a study with food handlers using structural equation modeling. Food research international. 2019;125:108637.

19. Adane M, Teka B, Gismu Y, Halefom G, Ademe M. Food hygiene and safety measures among food handlers in street food shops and food establishments of Dessie town, Ethiopia: a community-based cross-sectional study. PloS one. 2018;13(5):e0196919.

20. Makhunga S, Mashamba-Thompson TP, Hlongwa M, Hlongwana KW. Evidence on Food Control System in Charitable Food Assistance System: A Systematic Scoping Review. 2018.

21. Warshawsky DN. Urban Food Insecurity and the advent of food banking in southern Africa: African Food Security Urban Network (AFSUN); 2011.

22. Donkor ES, Kayang BB, Quaye J, Akyeh ML. Application of the WHO keys of safer food to improve food handling practices of food vendors in a poor resource community in Ghana. Int J Environ Res Public Health. 2009;6.

23. Five keys to safer food manual. Geneva: WHO; 2006.

24. da Vitoria AG, de Souza Couto Oliveira J, de Almeida Pereira LC, de Faria CP, de Sao Jose JFB. Food safety knowledge, attitudes and practices of food handlers: A cross-sectional study in school kitchens in Espirito Santo, Brazil. BMC Public Health. 2021;21(1):349.

25. Isoni Auad L, Cortez Ginani V, Stedefeldt E, Yoshio Nakano E, Costa Santos Nunes A, Puppin Zandonadi R. Food Safety Knowledge, Attitudes, and Practices of Brazilian Food Truck Food Handlers. Nutrients. 2019;11(8).

26. Mohlisi Mohd Asmawi U, Azureen Norehan A, Salikin K, Ain Syafiqah Rosdi N, Amira Thaqifah Abdul Munir N, Balqis Muhammad Basri N, et al. An Assessment of Knowledge, Attitudes and Practices in Food Safety Among Food Handlers Engaged in Food Courts. Current Research in Nutrition and Food Science Journal. 2018;6(2):346-53.

27. Ravinder EB, Saraswathi A. Literature Review Of Cronbach alpha coefficient (Α) And Mcdonald's Omega Coefficient (Ω). European Journal of Molecular & Clinical Medicine. 2020;7(6):2943-9.

28. Fariba R, Gholamreza JK, Saharnaz N, Ehsan H, Masoud Y. Knowledge, attitude, and practice among food handlers of semi-industrial catering: a cross sectional study at one of the governmental organization in Tehran. Journal of Environmental Health Science and Engineering. 2018;16(2):249-56.

For peer review only

For peer review only - http://bmjopen.bmj.com/site/about/guidelines.xhtml

29. Kunadu AP-H, Ofosu DB, Aboagye E, Tano-Debrah K. Food safety knowledge, attitudes and self-reported practices of food handlers in institutional foodservice in Accra, Ghana. Food Control. 2016;69:324-30.

30. Nee SO, Sani NA. Assessment of knowledge, attitudes and practices (KAP) among food handlers at residential colleges and canteen regarding food safety. Sains Malaysiana. 2011;40(4):403-10.

31. Sani NA, Siow ON. Knowledge, attitudes and practices of food handlers on food safety in food service operations at the Universiti Kebangsaan Malaysia. Food control. 2014;37:210-7.

32. Abdi AM, Amano A, Abrahim A, Getahun M, Ababor S, Kumie A. Food hygiene practices and associated factors among food handlers working in food establishments in the Bole Sub City, Addis Ababa, Ethiopia. Risk Management and Healthcare Policy. 2020;13:1861.

33. Pollard CM, Mackintosh B, Campbell C, Kerr D, Begley A, Jancey J, et al. Charitable Food Systems' Capacity to Address Food Insecurity: An Australian Capital City Audit. Int J Environ Res Public Health. 2018;15(6).

34. De Boeck E, Jacxsens L, Goubert H, Uyttendaele M. Ensuring food safety in food donations: Case study of the Belgian donation/acceptation chain. Food Res Int. 2017;100(Pt 2):137-49.

35. De Pieri B, Tallarico T, Baglioni S, Soler U, Ricciuti E. European Policy for Food Security: The Surplus Food Redistribution Option. Foodsaving in Europe2017. p. 13-35.

36. Montesclaros J, Caballero-Anthony M, Schlundt J. Food Safety in ASEAN: Pitfalls of Complacency. 2018.

37. Regan Á, McConnon Á, Holah J. Food Hygiene and Food Workers: From Complacency to Compliance. Handbook of Hygiene Control in the Food Industry: Elsevier; 2016. p. 197-203.

38. Adzoyi PN, Honyenuga BQ. Exploring Knowledge, Attitudes and Practices towards Food Hygiene by Hospitality Students in Ghana. Journal of Food Sci and quality mgmt. 2014;26:2225-0557.

39. Sharif L, Obaidat MM, Al-Dalalah M-R. Food hygiene knowledge, attitudes and practices of the food handlers in the military hospitals. Food and Nutrition Sciences. 2013;4(03):245.

40. Al-Shabib NA, Mosilhey SH, Husain FM. Cross-sectional study on food safety knowledge, attitude and practices of male food handlers employed in restaurants of King Saud University, Saudi Arabia. Food Control. 2016;59:212-7.

41. Asmawi UMM, Norehan AA, Salikin K, Rosdi NAS, Munir NATA, Basri NBM. An assessment of knowledge, attitudes and practices in food safety among food handlers engaged in food courts. Current Research in Nutrition and Food Science Journal. 2018;6(2):346-53.

For peer review only

For peer review only - http://bmjopen.bmj.com/site/about/guidelines.xhtml

42. Bou-Mitri C, Mahmoud D, El Gerges N, Abou Jaoude M. Food safety knowledge, attitudes and practices of food handlers in lebanese hospitals: A cross-sectional study. Food control. 2018;94:78-84.

43. El-Nemr I, Mushtaha M, Irungu P, Asim H, Tang P, Hasan M, et al. Assessment of food safety knowledge, self-reported practices, and microbiological hand hygiene levels of produce handlers in Qatar. Journal of food protection. 2019;82(4):561-9.

For peer review only

For peer review only - http://bmjopen.bmj.com/site/about/guidelines.xhtml

STROBE Statement—Checklist of items that should be included in reports of ***cross-sectional studies***

	Item No	Recommendation
**Title and abstract**	1	(*a*) Indicate the study's design with a commonly used term in the title or the abstract
		(*b*) Provide in the abstract an informative and balanced summary of what was done and what was found
**Introduction**		
Background/rationale	2	Explain the scientific background and rationale for the investigation being reported
Objectives	3	State specific objectives, including any prespecified hypotheses
**Methods**		
Study design	4	Present key elements of study design early in the paper
Setting	5	Describe the setting, locations, and relevant dates, including periods of recruitment, exposure, follow-up, and data collection
Participants	6	(*a*) Give the eligibility criteria, and the sources and methods of selection of participants
Variables	7	Clearly define all outcomes, exposures, predictors, potential confounders, and effect modifiers. Give diagnostic criteria, if applicable
Data sources/ measurement	8*	For each variable of interest, give sources of data and details of methods of assessment (measurement). Describe comparability of assessment methods if there is more than one group
Bias	9	Describe any efforts to address potential sources of bias
Study size	10	Explain how the study size was arrived at
Quantitative variables	11	Explain how quantitative variables were handled in the analyses. If applicable, describe which groupings were chosen and why
Statistical methods	12	(*a*) Describe all statistical methods, including those used to control for confounding
		(*b*) Describe any methods used to examine subgroups and interactions
		(*c*) Explain how missing data were addressed
		(*d*) If applicable, describe analytical methods taking account of sampling strategy
		(*e*) Describe any sensitivity analyses
**Results**		
Participants	13*	(a) Report numbers of individuals at each stage of study—eg numbers potentially eligible, examined for eligibility, confirmed eligible, included in the study, completing follow-up, and analysed
		(b) Give reasons for non-participation at each stage
		(c) Consider use of a flow diagram
Descriptive data	14*	(a) Give characteristics of study participants (eg demographic, clinical, social) and information on exposures and potential confounders
		(b) Indicate number of participants with missing data for each variable of interest
Outcome data	15*	Report numbers of outcome events or summary measures
Main results	16	(*a*) Give unadjusted estimates and, if applicable, confounder-adjusted estimates and their precision (eg, 95% confidence interval). Make clear which confounders were adjusted for and why they were included
		(*b*) Report category boundaries when continuous variables were categorized
		(*c*) If relevant, consider translating estimates of relative risk into absolute risk for a meaningful time period
Other analyses	17	Report other analyses done—eg analyses of subgroups and interactions, and sensitivity analyses

For peer review only - http://bmjopen.bmj.com/site/about/guidelines.xhtml

**Discussion**		
Key results	18	Summarise key results with reference to study objectives
Limitations	19	Discuss limitations of the study, taking into account sources of potential bias or imprecision. Discuss both direction and magnitude of any potential bias
Interpretation	20	Give a cautious overall interpretation of results considering objectives, limitations, multiplicity of analyses, results from similar studies, and other relevant evidence
Generalisability	21	Discuss the generalisability (external validity) of the study results
**Other information**		
Funding	22	Give the source of funding and the role of the funders for the present study and, if applicable, for the original study on which the present article is based

*Give information separately for exposed and unexposed groups.

Note: An Explanation and Elaboration article discusses each checklist item and gives methodological background and published examples of transparent reporting. The STROBE checklist is best used in conjunction with this article (freely available on the Web sites of PLoS Medicine at http://www.plosmedicine.org/, Annals of Internal Medicine at http://www.annals.org/, and Epidemiology at http://www.epidem.com/). Information on the STROBE Initiative is available at www.strobe-statement.org.

For peer review only
